# Editorial: Highlights in psycho-oncology: study protocols - improving evidence for future personalised cancer care

**DOI:** 10.3389/fpsyg.2024.1422054

**Published:** 2024-08-01

**Authors:** Gregor Weissflog, Simon Dunne

**Affiliations:** ^1^Department of Medical Psychology and Medical Sociology, University Medical Center, Leipzig, Germany; ^2^Comprehensive Cancer Center Central Germany (CCCG) Leipzig-Jena, Leipzig, Germany; ^3^Faculty of Science and Health, School of Psychology, Dublin City University, Dublin, Ireland

**Keywords:** cancer, psycho-oncology, cancer survivorship, study protocol, research methods

## Past, present, future and the quality of research in psycho-oncology

Research in the field of psycho-oncology has developed steadily over the past 40 years. Key studies have been conducted since then with a focus on improving the psychosocial care of cancer patients and their families and friends (e.g., Kissane, 2022). In recent years, this has expanded to include the use of technology-assisted psychosocial interventions, such as the use of Artificial Intelligence and Large Language models (LLMs), in the care of individuals affected by cancer. Chatbots, virtual reality goggles and active video games are examples of more recently developed psycho-oncological interventions for cancer patients (Holohan and Fiske, 2021; Zhang et al., 2022). To further strengthen scholarship in this field and help develop effective real world assessments and interventions aimed at delivering optimal personalized cancer care, future research has to fulfill strict research quality criteria. These can be implemented in the form of stringent and public available study protocols. The question may arise as to why we should endeavor to produce high-quality study protocols. This will be explained briefly below.

## Study protocols strengthen research designs and provide important opportunities for researchers

By allowing for methods and processes of a research study to be evaluated by the peer review process in advance, study protocols provide important opportunities for researchers. In particular, receiving external expert opinion on the methods of a study can enable researchers to enhance their study designs and reduce the amount of confounding variables in a study. Published protocols are also valuable to researchers as they can give funding agencies confidence in a research proposal, thereby prioritizing funding of the research (Eysenbach, 2004). Timely published protocols can also prevent other reseachers unneccesarily duplicating research designs (e.g., that are based on theory). Rather than preventing researchers from pursuing a research idea, the sharing of research ideas and methods opens the door for potential national and international collaboration with like-minded researchers. Sharing research ideas and methods in this way is also in keeping with the “shared” characteristic of Open Science (Thibault et al., 2023).

## Study protocols limit the effect of bias

The use of study protocols can potentially minimize the impact of different biases on *real* empirical evidence. Among these are publication biases, confirmation biases and researchers engaging in questionable research practices. Publication bias refers to the failure of journals to publish studies (including clinical trials) with non-significant findings. This can be linked to a confirmation bias (Nickerson, 1998); in other words, uncritically accepting (and believing) significant findings that were expected but critically scrutinizing (and disbelieving) non-significant findings. In this context, it is common for reviewers/editors to indicate that non-significant findings result from methodological flaws, even though the methodology used may instead have been more robust than the approach of previously published studies. There may be subjective concerns as well, relating to those reviewers who “have a stake in” an approach or methodology. As indicated by Ioannidis (2005, p. 0698): “Prestigious investigators may suppress via the peer review process the appearance and dissemination of findings that refute their findings, thus condemning their field to perpetuate false dogma”. The pressure to publish significant (and novel) findings is particularly problematic, as it has been found to perpetuate “questionable research practices” in psychology. This includes, among others, the following issues: HARKing: Hypothesizing After Results are Known (or: presenting exploratory findings as confirmatory findings), “Peeking” (collecting extra cases until significance is reached; not conforming to pre-determined sample size), removing key variables in order to manipulate findings, or, in more extreme cases, data falsification (Vermeulen and Hartmann, 2015). In contrast, a peer-reviewed protocol ensures that there is no bias relating to the results of the study—the merits of the methodological approach are evaluated in the absence of results. This helps to avoid confirmation bias or other related biases.

## This special Research Topic

The present Research Topic aimed to collect recent study protocols from the field of psycho-oncology that address current research questions and have the potential to inform future directions of psycho-oncology care and research. A further aim was to highlight studies that will be conducted on different types of cancer, stages of the disease, and with groups currently understudied and underserved.

This Research Topic contains proposed work from Europe (Portugal, Spain and Germany), America (United States and Canada) and Asia (China). Unfortunately, we could not include African or Australian research perspectives. In total, there are seven study protocols and one mini review.

## Protocols on interventions in psycho-oncology

Three main themes are addressed by the contributions to this Research Topic. The first theme, *interventions in psycho-oncology*, includes study protocols targeting multidisciplinary cancer care teams as well as interventions in cancer patients, such as eHealth applications and dyadic interventions. A study protocol by Chênevert et al. aims to implement and evaluate a participatory interventional approach that fosters team resilience in multidisciplinary cancer care. García-Torres et al. present their protocol in order to investigate the efficacy of fostering psychological flexibility in cancer patients by comparing different modes of an Acceptance and Commitment Therapy intervention (face-to-face+app vs. face-to-face only) with a waitlist control group in a mixed cancer sample. Waldman et al. propose a study to investigate the utility of a supportive care app for improving symptom management and enhancing quality of life and adaptive coping in advanced non-small cell lung cancer patients. Finally, Song et al. complete the first theme with their scoping review (not a study protocol!). They highlight poor empirical evidence regarding the effectiveness of dyadic-based physical activity interventions in improving cancer-related fatigue in cancer survivors.

## Protocols on the assessment of unique views and perspectives of cancer-affected persons

The second theme developed in this Research Topic is *ecological validity* within the field of psycho-oncology. It includes the qualitative assessment of very personal insights from cancer survivors and their caregivers, in this case from a Portuguese perspective. A first study by Fernandes, Domingos, Castro et al. aims to explore the needs and expectations of family caregivers of cancer patients in palliative care. A second study developed by the same working group (Fernandes, Domingos, Almeida et al.) intends to explore enablers, barriers, and strategies to build resilience among cancer survivors by conducting qualitative interviews with cancer survivors and healthcare professionals.

## Protocols on precision assessment of cancer-related burdens

The third and final theme deals with *internal validity* in psycho-oncology. This includes the thorough assessment of the prevalence of mental disorders, psychosocial distress, and perceived need for psychosocial support in cancer patients and their relatives, as well as the development and psychometric testing of a pediatric chronic graft-vs.-host disease symptom scale. Mehnert-Theuerkauf et al. present a large scale prospective multi-center observational cohort design from Germany with longitudinal data across four time points [within 2 months after cancer diagnosis (t1)], and half yearly follow-ups up to 18 months after diagnosis. Their main outcomes will be the prevalence of mental disorders and psychosocial distress as well as the perceived need for psychosocial support in cancer patients and their relatives. Mitchell et al. inform about their plans to develop a psychometrically valid pediatric cGVHD Symptom Scale (PCSS) and a companion caregiver-proxy measure to capture the symptom burden experienced by children with cGVHD in a multi-center, two-phase protocol.

## Future directions

To conclude, the current Research Topic highlights the value of study protocols in psycho-oncology. As we have outlined, study protocols have the potential to enhance study designs through expert peer review, reduce confounding variables, minimize the impact of biases and questionable research practices in psycho-oncology research and may help to produce research that is of better quality and in line with the principles of Open Science. We finish with a quote from Prof Brian M Hughes, who suggests the following idealized future scenario about study protocols in his book “Psychology in Crisis”: “in a reimagined journal system authors would pre-register their intention to conduct a specific study, at which point their proposed methods would be formally peer-reviewed. Later, they would submit a partial report of the study along with the study's dataset. The journal would recruit new peer reviewers to recommend final publication on the basis of the methods described, prior to knowing the results. The journal could ask separate reviewers to analyse the dataset according to the pre-registered protocol, to confirm the authors' own results. The final manuscript would be reviewed in the traditional fashion. Assuming revisions are requested and carried out, the second draft would be reviewed by fresh reviewers. Ultimately the paper would be published along with a note listing all the various reviewers who have been involved (Hughes, 2018, p. 170)”. We should add that the peer review process needs to consider and protect against error and poor quality reviews associated with the current use of LLM-generated “peer review” reports, which are at risk of conflating different sources of information and evaluating articles on the basis of inaccurate and misleading information (Brod and Widyadari, 2023). Although there is much work to do before a peer review system which accommodates these changes is possible. The protocols described in this Research Topic fulfill these criteria by providing a priori differentiated and detailed information on their questions or hypotheses, research methodology and planned analyses.

## Best practice in writing study protocols

Researchers who are interested in designing studies and writing protocols can access the information disseminated by the *Equator-Network*: https://www.equator-network.org/. This website provides an overview of necessary information dependent on the planned research. It presents specific reporting guidelines for different research designs, such as SPIRIT (Chan et al., 2013), CONSORT (Schulz et al., 2010) and CONSERVE (Orkin et al., 2021) or STROBE (Vandenbroucke et al., 2007). Adhering to these guidelines can lead to research of better quality, or, in other words, “more structure, less ‘Wuthering Heights”' (Treweek, 2019).

## Author contributions

GW: Writing – original draft, Writing – review & editing. SD: Writing – original draft, Writing – review & editing.
